# Supplementary material for: Transposable elements may enhance antiviral resistance in HIV-1 elite controllers
Source: bioRxiv. 2023 Dec 12:2023.12.11.571123. Preprint. [Version 1] doi: 10.1101/2023.12.11.571123 (PMC10760019; doi:10.1101/2023.12.11.571123)
Supplement: 8 [file NIHPP-2023.12.11.571123V1-supplement-1.pdf]

bioRxiv preprint doi: https://doi.org/10.1101/2023.12.11.571123; this version posted December 12, 2023. The copyright holder for this preprint (which was not certified by peer review) is the author/funder, who has granted bioRxiv a license to display the preprint in perpetuity. It is made available under aCC-BY 4.0 International license.

Supplemental Figures

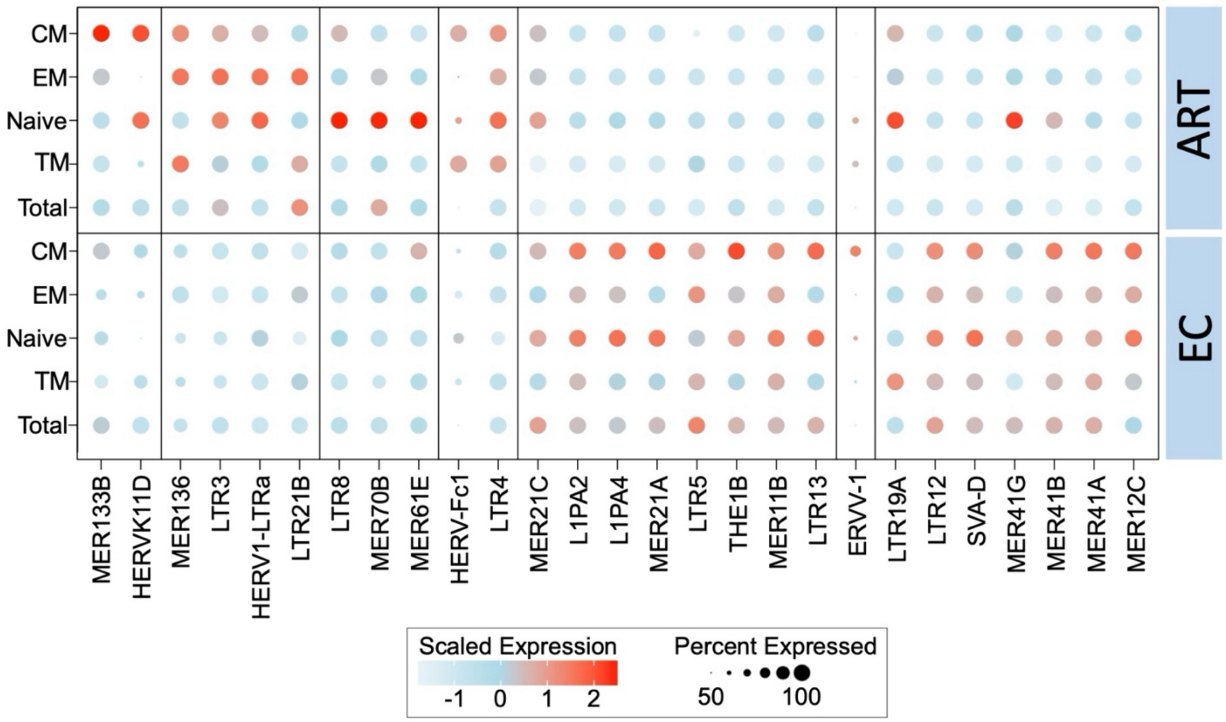

Supplementary Figure 1. Unique retrotranscriptome of ECs vs PLWH-on-ART

Dot plot illustrating the intensity and abundance of the differential expression of TE families between ECs and PLWH-on-ART, separated by CD4$^+$ T cell subtype. Coloration is scaled from lower (white) to higher (red) expression. Point size is directly proportional to the percentage of that sample group expressing the given TE family. Data source: Jiang et al., 2020.

bioRxiv preprint doi: https://doi.org/10.1101/2023.12.11.571123; this version posted December 12, 2023. The copyright holder for this preprint (which was not certified by peer review) is the author/funder, who has granted bioRxiv a license to display the preprint in perpetuity. It is made available under aCC-BY 4.0 International license.

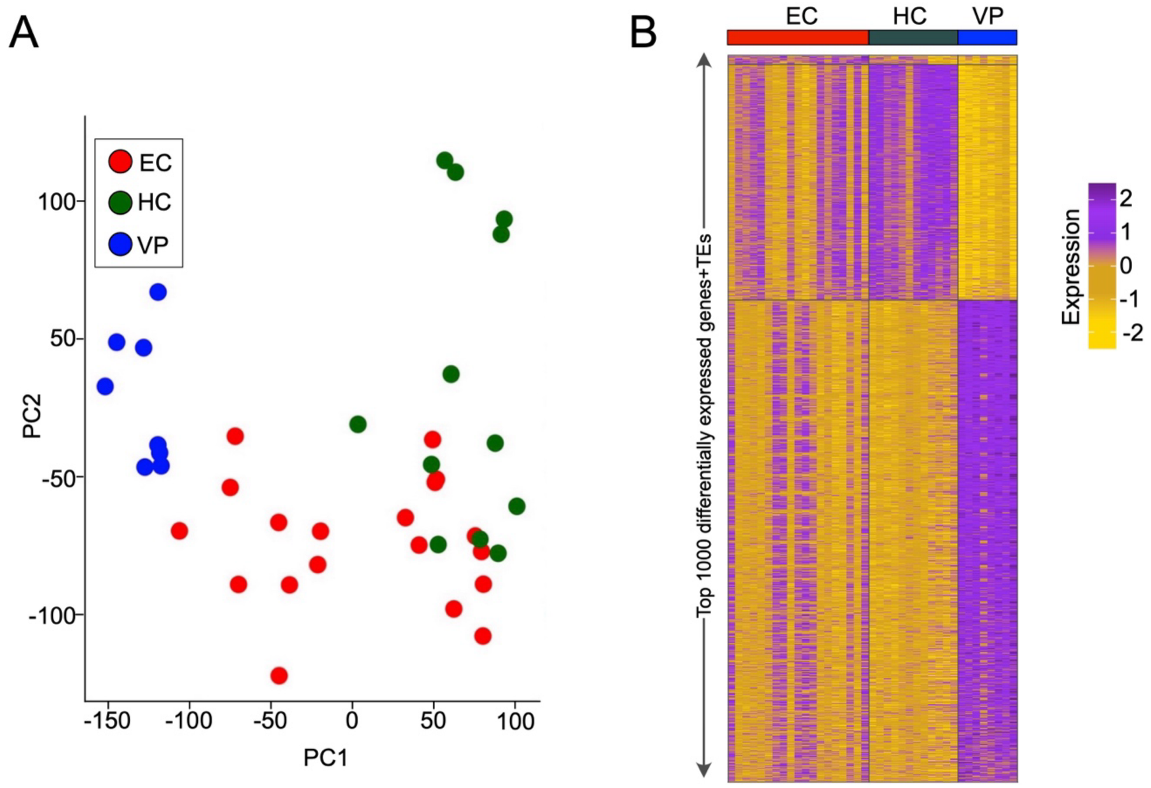

Supplementary Figure 2. Heterogeneity of EC (retro)transcriptome

A. PCA plot from PBMCs of ECs (red), VPs (blue), and HCs (green), based on the most variably expressed genes and TE families. Every dot is a PBMC sample from an individual. Data source: Zhang et al., 2018.

B. Heatmap displaying the scaled expression of genes and TEs distinguishing EC, HC, and VP samples shown in the previous plot. Every row denotes a gene or retroelement. Data source: Zhang et al., 2018.

bioRxiv preprint doi: https://doi.org/10.1101/2023.12.11.571123; this version posted December 12, 2023. The copyright holder for this preprint (which was not certified by peer review) is the author/funder, who has granted bioRxiv a license to display the preprint in perpetuity. It is made available under aCC-BY 4.0 International license.

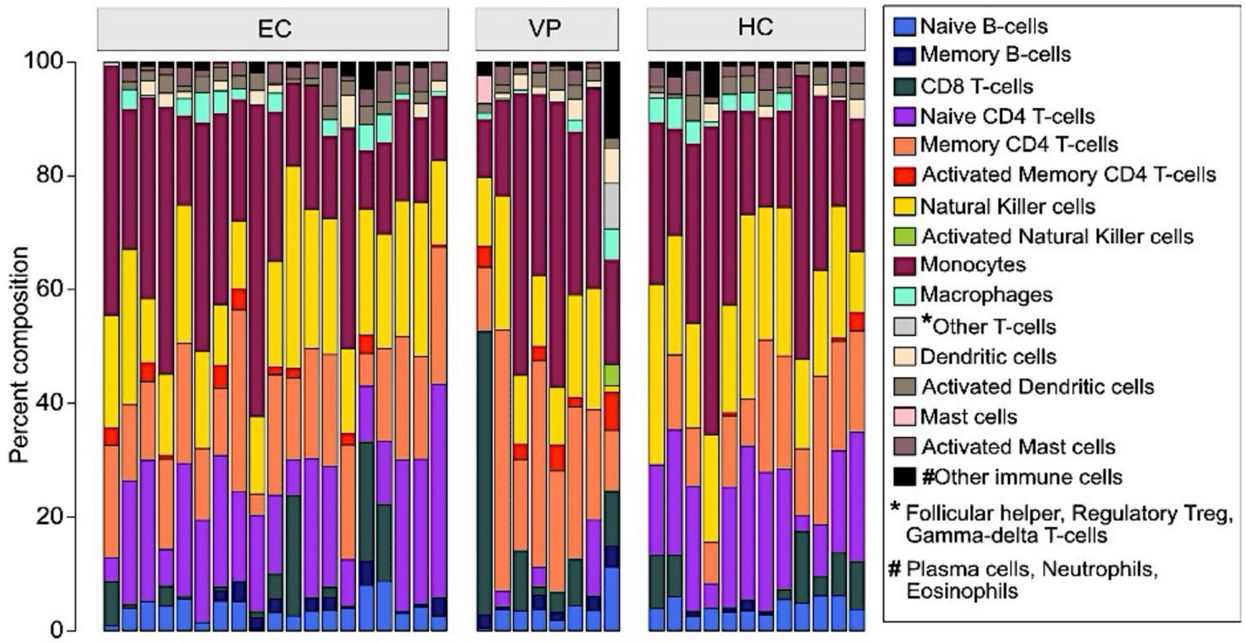

Supplementary Figure 3. Immune cell profiles of ECs, VPs, and HCs

Stacked barplot showing the immune cell composition of ECs, VPs, and HCs from the deconvolution analysis of PBMC RNA-seq data. Data source: Zhang et al., 2018.

bioRxiv preprint doi: https://doi.org/10.1101/2023.12.11.571123; this version posted December 12, 2023. The copyright holder for this preprint (which was not certified by peer review) is the author/funder, who has granted bioRxiv a license to display the preprint in perpetuity. It is made available under aCC-BY 4.0 International license.

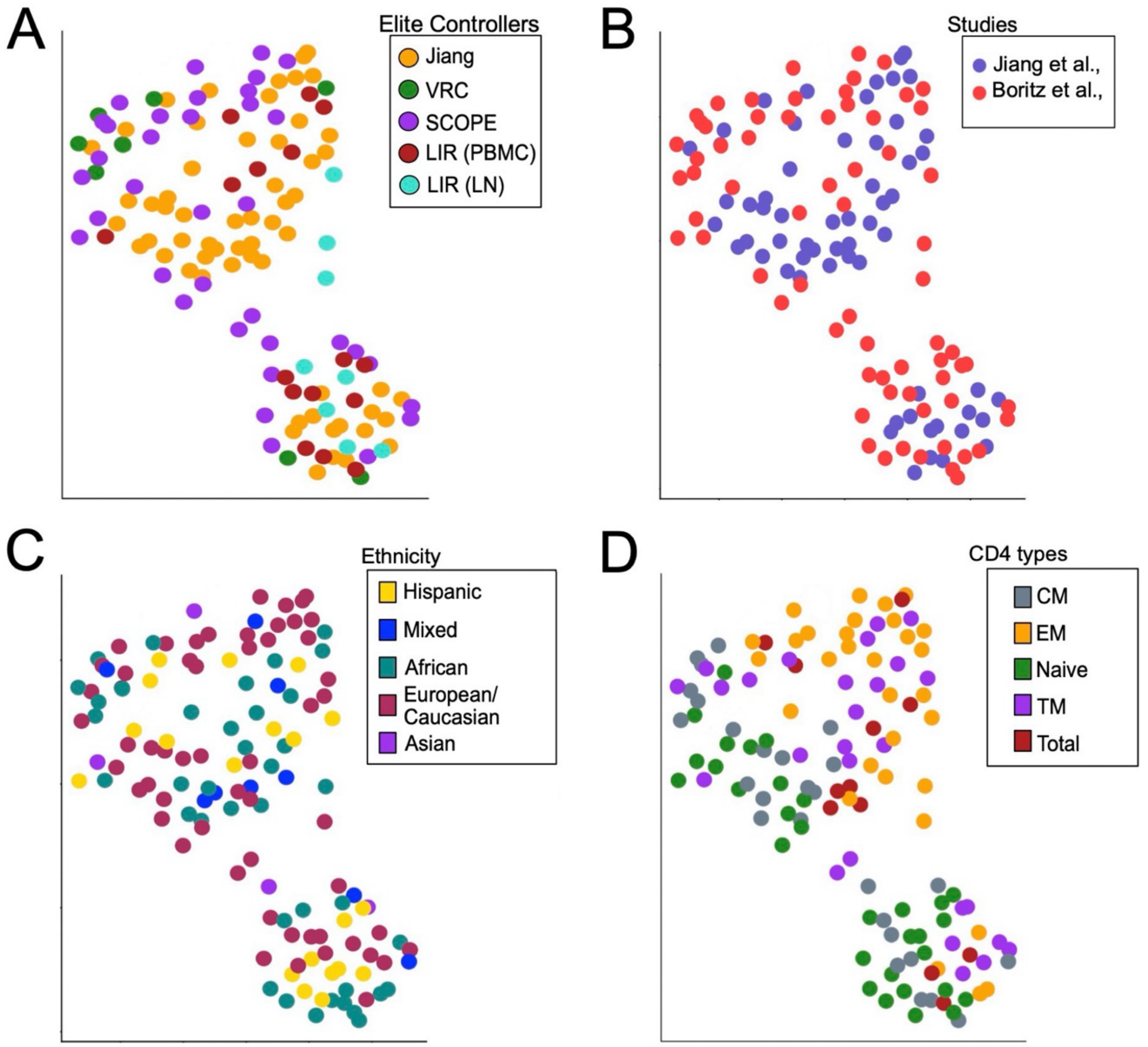

Supplementary Figure 4. Characterization of the EC clusters

A. UMAP plot from Figure 3A with samples color coded based on recruitment location of each patient. Data sources: Jiang et al., 2020 and Boritz et al., 2016.

B. UMAP plot from Figure 3A with samples color coded based on the corresponding source study. Data sources: Jiang et al., 2020 and Boritz et al., 2016.

C. UMAP plot from Figure 3A with samples color coded based on patient ancestry, inferred by HapMap to make direct variant comparisons. Data sources: Jiang et al., 2020 and Boritz et al., 2016.

D. UMAP plot from Figure 3A with samples color coded based on the $CD4^+$ subtypes: naïve, central memory (CM), transitional memory (TM), effector memory (EM), and total. Data sources: Jiang et al., 2020 and Boritz et al., 2016.

bioRxiv preprint doi: https://doi.org/10.1101/2023.12.11.571123; this version posted December 12, 2023. The copyright holder for this preprint (which was not certified by peer review) is the author/funder, who has granted bioRxiv a license to display the preprint in perpetuity. It is made available under aCC-BY 4.0 International license.

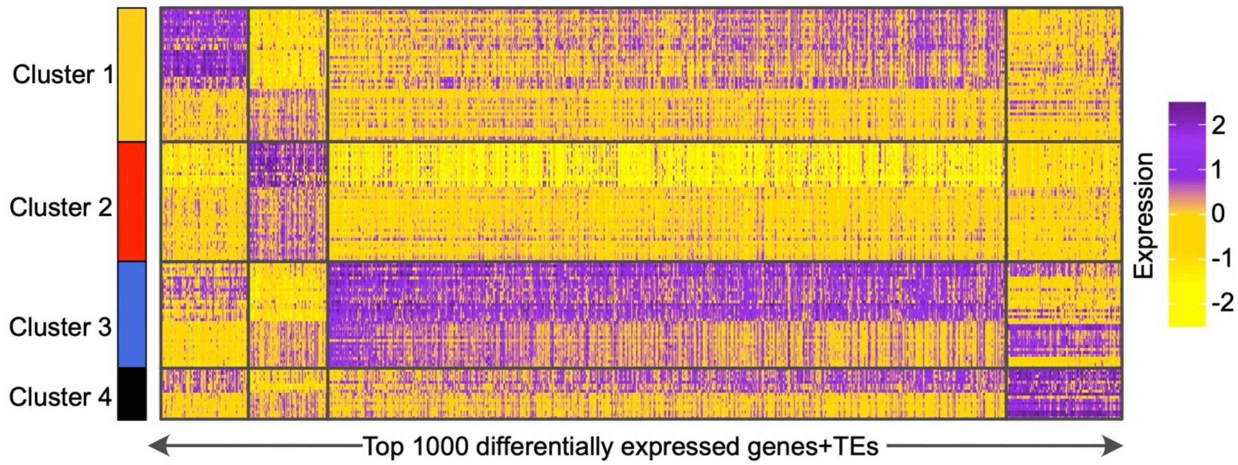

Supplementary Figure 5. Broad (retro)transcriptomic profile of the EC clusters

Heatmap of the differentially expressed genes (DEGs) that distinguish the EC clusters from each other. Data source: Jiang et al., 2020 and Boritz et al., 2016.

bioRxiv preprint doi: https://doi.org/10.1101/2023.12.11.571123; this version posted December 12, 2023. The copyright holder for this preprint (which was not certified by peer review) is the author/funder, who has granted bioRxiv a license to display the preprint in perpetuity. It is made available under aCC-BY 4.0 International license.

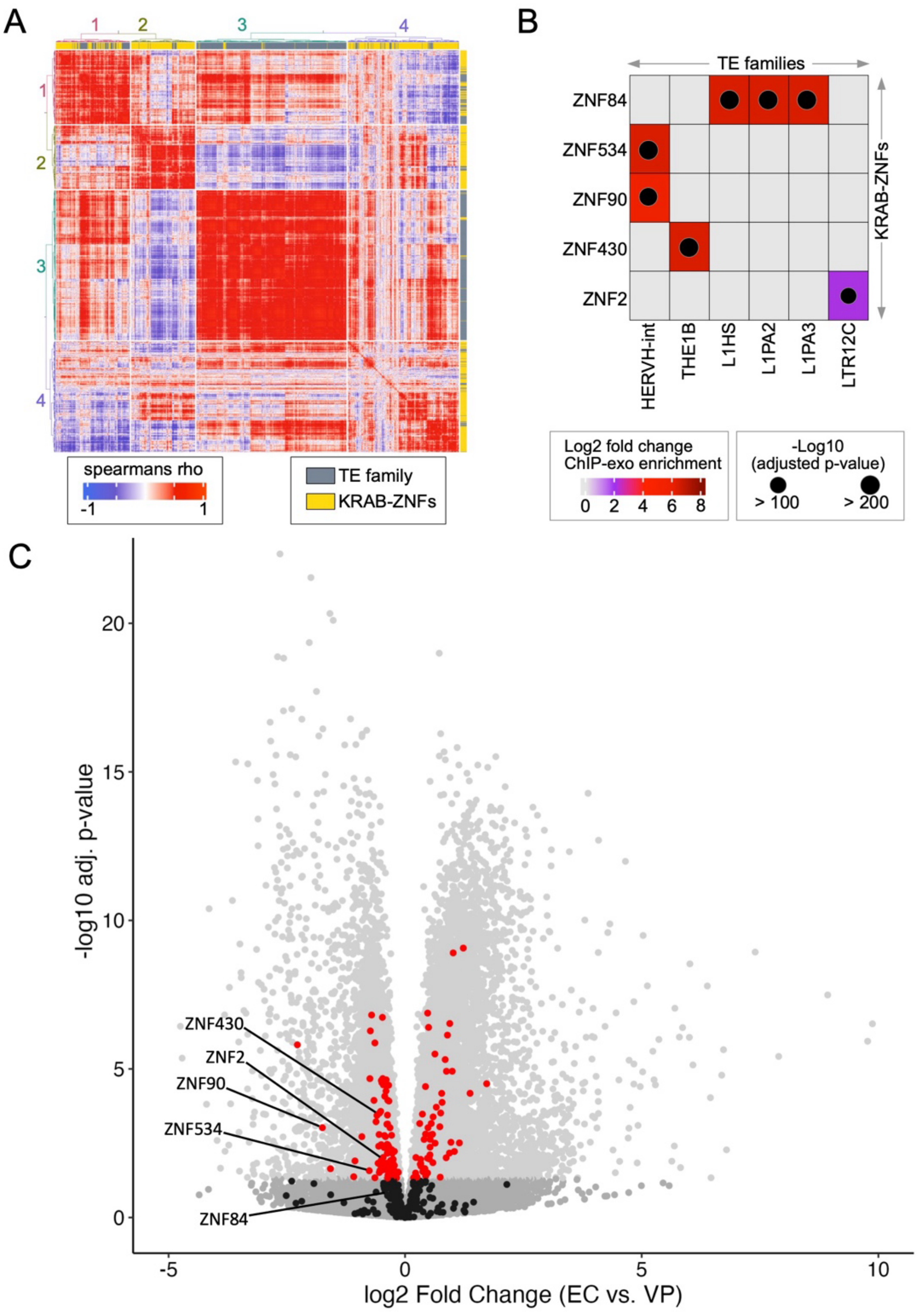

Supplementary Figure 6. KZNF dynamics in ECs

A. Heatmap showing the correlation matrix of TE families with KZNFs across the analyzed EC samples. Clustered pairwise correlation matrix generated by weighted gene co-expression network analysis, using the Spearman's rank correlation & Euclidean distance. Data sources: Jiang et al., 2020 and Boritz et al., 2016.

bioRxiv preprint doi: https://doi.org/10.1101/2023.12.11.571123; this version posted December 12, 2023. The copyright holder for this preprint (which was not certified by peer review) is the author/funder, who has granted bioRxiv a license to display the preprint in perpetuity. It is made available under aCC-BY 4.0 International license.

B. Heatmap showing the enrichment for binding of a subset of KZNFs to TE families with increased expression in ECs. Coloration is scaled from lower (gray) to higher (red) ChIP-exo enrichment in HEK cells. Point size is directly proportional to the -log10-transformed adjusted p-value. Data source: Imbeault et al., 2017.

C. Volcano plot displaying the differential expression of genes between EC and VP samples. KZFP genes are in red (p-value < 0.05) or black (p-value > 0.05). All other genes are in gray. Data source: Zhang et al., 2018.
